# Association of Sociodemographic Factors with Physical Activity and Sleep Quality in Arab and Non-Arab Individuals of Both Sexes during the COVID-19 Pandemic

**DOI:** 10.3390/healthcare11152200

**Published:** 2023-08-04

**Authors:** Ashokan Arumugam, Danya Murat, Asma Javed, Sara Atef Ali, Ibrahim Mahmoud, Khaled Trabelsi, Achraf Ammar

**Affiliations:** 1 Department of Physiotherapy, College of Health Sciences, University of Sharjah, Sharjah P.O. Box 27272, United Arab Emirates; ashokanpt@gmail.com (A.A.);; 2 Neuromusculoskeletal Rehabilitation Research Group, RIMHS—Research Institute of Medical and Health Sciences, University of Sharjah, Sharjah P.O. Box 27272, United Arab Emirates; 3 Sustainable Engineering Asset Management Research Group, RISE—Research Institute of Sciences and Engineering, University of Sharjah, Sharjah P.O. Box 27272, United Arab Emirates; 4 Department of Physiotherapy, Manipal College of Health Professions, Manipal Academy of Higher Education, Manipal 576104, Karnataka, India; 5 Department of Family Medicine and Behavioural Sciences, College of Medicine, University of Sharjah, Sharjah P.O. Box 27272, United Arab Emirates; iabdelmahmoud@sharjah.ac.ae; 6 High Institute of Sport and Physical Education of Sfax, University of Sfax, Sfax 3000, Tunisia; 7 Department of Training and Movement Science, Institute of Sport Science, Johannes Gutenberg-University Mainz, 55128 Mainz, Germany

**Keywords:** COVID-19, pandemic, physical activity, sedentary behavior, sleep quality

## Abstract

We explored the association of sociodemographic and anthropometric factors with self-reported physical activity (PA) and sleep quality in Arab and non-Arab individuals of both sexes during the COVID-19 pandemic. In this cross-sectional study, 638 participants (those recovered from COVID-19 = 149, and non-infected = 489) of both sexes aged 18–55 years were recruited. Their sociodemographic and anthropometric information, PA (self-reported using the International Physical Activity Questionnaire Short-form [IPAQ-SF)]) and sleep quality (self-reported using the Pittsburgh Sleep Quality Index [PSQI]) were documented. The association between participants’ characteristics, PA levels, and sleep quality were determined using the chi-squared test. Variables significantly associated with IPAQ and PSQI in bivariate analyses were included in a multivariate binary logistic regression model. Men were more active than women (odds ratio [OR] = 1.66, *p* = 0.010), and non-Arab participants were more active than Arab ones (OR = 1.49, *p* = 0.037). Participants ≥40 years, men, non-Arab participants, and those who were working were more likely to have a good sleep quality than those ≤40 years (OR 1.70, *p* = 0.048), women (OR 1.10, *p* = 0.725), Arab individuals (OR 1.95, *p* = 0.002), and unemployed people (OR 2.76, *p* = 0.007). Male and non-Arab participants seemed to have a better self-reported PA and sleep quality compared to female and Arab participants, during the pandemic.

## 1. Introduction

The coronavirus disease 2019 (COVID-19) pandemic was found to impact everyday life worldwide [1]. Individuals who were economically vulnerable and faced a low risk of serious illness might not follow the recommendations and instructions to engage in protective behaviors, such as keeping socially distant, and wearing a mask, therefore putting vulnerable individuals in danger of becoming infected with COVID-19, which would lead to a prolonged pandemic [2]. During the pandemic, social distance measures were implemented, including the closure of schools, non-essential businesses, gyms, playgrounds, and public swimming pools, so that people would stay home [3].

Unfortunately, the social distancing measures led to a decrease in moderate-to-vigorous intensity physical activity (PA), and an increase in sedentary behavior, among individuals [4,5]. The World Health Organization recommends that adults perform 150–300 min of moderate-intensity PA, 75–150 min of vigorous-intensity PA, or an equivalent combination of moderate and vigorous intensity PA per week [6]. However, a high prevalence of sedentary behavior and physical inactivity has been reported among the United Arab Emirates (UAE) population [7,8]. There was a rise in unhealthy lifestyle behaviors, including but not limited to, decreased physical activity (30%) and decreased sleep (20.8%) during the COVID-19 pandemic in the UAE [9].

The physical benefits of PA include a healthy body weight, musculoskeletal and cardiovascular health, and neuromuscular awareness for coordination and controlling movement [10]. PA also has some psychological benefits, including building self-esteem, managing anxiety, and more [10]. PA is considered to be one of the factors influencing social wellbeing [11]. Since the social interactions and daily activities of individuals were restricted, the prevalence of psychological disorders (such as anxiety and depression) was widely reported during quarantine [12].

A study conducted on physiotherapy professionals and students during COVID-19 concluded that there was a significant reduction in self-reported PA and energy expenditure levels (using the International Physical Activity Questionnaire Short-form (IPAQ-S)) [13]. Another study evaluated, using the IPAQ-S, how the self-reported PA and sedentary time changed during lockdown in the Spanish population, and concluded that there was a decrease in daily PA, and an increase in sedentary time, among the Spanish adult population, especially among young people, students, and very active men, during the COVID-19 lockdown [14].

A cross-sectional study performed on university students during the COVID-19 pandemic showed that there was a decrease in the time spent on PA, a change in the type and level of PA, and an increase in sitting time and sedentary behavior among female and male students [15]. Men older than 26 years and living in a household with parents with a low level of education were considered at risk of being physically less active [15]. Another study noted that women, who previously had a lower level of PA than men, showed a lower tendency to decrease during lockdown, which may reflect a greater resilience than men [16]. On the contrary, men have been found to have had a better wellbeing perception and PA rates, compared to women, during the COVID-19 lockdown period [11].

A systematic review of 14 studies revealed that moderate-intensity PA appears to be more effective in improving sleep quality compared to vigorous-intensity PA. Moreover, moderate-intensity PA has been found to have beneficial effects on sleep quality in all age groups in a healthy population [17]. Further, a meta-analysis substantiated that physical activity/exercise is a potential intervention to improve self-perceived and objective sleep metrics in individuals with and without sleep problems [18]. Therefore, it is important to assess the PA levels and sleep quality of individuals during the COVID-19 pandemic.

Psychological problems that increased during lockdown may potentially interfere with sleep patterns, and life in general, because of the prolonged and stressful COVID-19 pandemic [12,19,20]. The National Sleep Foundation recommends 7 to 9 h of sleep for adults, and 7 to 8 h of sleep for older adults [21]. Sleep has been identified as a vital component of physical, cognitive, and emotional health [22], with insufficient sleep showing associations with high mortality and morbidity [23]. Sleep quality has been found to diminish across a lifespan, with the most substantial influence observed on sleep efficiency (the ratio of total sleep-time to time in bed) [24]. Moreover, a poor sleep quality has been found to be associated with insufficient physical activity [25]. Poor sleep quality and sleep-pattern deviations are associated with increased risks of respiratory, cardiovascular, and cognitive diseases, as well as metabolic problems, mortality, and a poor quality of life [12,26]. Therefore, insufficient sleep, and its consequences on health, create a significant burden on the economic and healthcare systems [22].

A study on sleep quality and health during the COVID-19 outbreak using the Pittsburgh Sleep Quality Index (PSQI), a modified version of the Epworth Sleepiness Scale (ESS), and the Satisfaction, Alertness, Timing, Efficiency, and Duration questionnaire (SATED) concluded that the COVID-19 outbreak events were associated with a decreased sleep quality, and an increased negative mood, during the pandemic [27]. Furthermore, during the COVID-19 lockdown, an overall increase in anxiety, and a decrease in sleep quantity and quality were reported, with a specific sex difference in perceived anxiety (higher in females) [28]. The COVID-19 pandemic increased the chances of sleep disturbance, and affected the immune system function [29]. Alterations in sleep quality and patterns may lead to an impaired immune system, which is critical in the development and progression of COVID-19 [30].

A recent study has reported low levels of PA in young adults in the United Arab Emirates (UAE), below the minimum recommended levels required for the optimal functioning of the cardiorespiratory system [8]. Physical inactivity has been reported to be higher among women and Arab people, compared to men and non-Arab people, respectively, according to a compendium of physical inactivity prevalence in 38 Muslim countries [31].

Sex differences in PA and sleep have been reported in the literature. Some of the reasons for the decreased engagement in PA among women are cultural/societal barriers, the hot weather conditions in the UAE making it difficult to exercise outdoors, restrictive traditional attire, lack of family support, etc. [32,33]. There was a need to encourage women to spend more time on physical activity at a vigorous rate during the COVID-19 pandemic, considering their low physical activity levels [11]. A higher prevalence of depression, susceptibility to emotional exhaustion, stress, and associated adverse health effects in women, and sex-based differences in the biology of sleep, might be some reasons for the differences in sleep quality between men and women [24,34].

Obtaining current data regarding the PA levels and sleeping behavior during the pandemic, and investigating the possible sociodemographic moderators, is crucial to ensure data-driven targeted measures to mitigate lockdown impact, and improve our preparedness for future pandemics. The aim of this study was to explore the association of sociodemographic and anthropometric factors with self-reported PA and sleep quality in Arab and non-Arab individuals of both sexes, during the COVID-19 pandemic. We hypothesized that PA levels and sleep quality would be low/poor in Arab participants compared to non-Arab ones, and in women compared to men, in the UAE [9,31,34,35,36].

## 2. Materials and Methods

### 2.1. Study Design and Participants

A retrospective design was used to investigate PA levels and sleep quality in adults during the COVID-19 pandemic. Ethical approval for this study was obtained from the Research Ethics Committee of the University of Sharjah (REC-21-03-07-02-S).

Arab and non-Arab individuals of both sexes, recovered from, or not infected with COVID-19, aged between 18 and 55 years, were recruited, using a convenient sampling method. Participants were recruited through posts on social media websites, university/school announcements, and word-of-mouth advertising. The exclusion criteria included individuals who currently had a COVID-19 infection or long-COVID symptoms, and those with other comorbidities and/or taking medications that might affect their PA and/or sleep. In addition, severe or critical COVID-19 cases (diagnosed before or at the time of the study) with acute respiratory distress syndrome, sepsis, septic shock, respiratory failure, or multi-organ failure were excluded. Furthermore, those with any prior history of any musculoskeletal, rheumatic, cardiorespiratory, or systemic diseases, or recent surgeries affecting their sleep quality, PA levels, or daily living activities, were excluded.

The sample size for this study was calculated to be a minimum of 500, based on a formula for cross-sectional study design {n = [Z^2^ P (1 − P)]/d^2^} plus a 30% non-response rate. The following parameters were used to estimate the sample size (n): 95% confidence level (Z = 1.96), prevalence (P), and a marginal error (d) of 5%.

### 2.2. Study Variables

A questionnaire with four sections was prepared online, using Google Forms, and sent to participants through text messages and social media platforms, to document their responses. The questionnaire was available in English and Arabic versions. The first section included information about the study’s purpose and procedures, along with an informed consent request. Upon reading the study protocol and providing their informed consent, participants were able to proceed with the study questionnaire. The second section included questions related to sociodemographic/anthropometric information regarding their age, sex, body weight (kg), height (cm), nationality, occupation, number of people in their house, number of people per room, history of smoking, and COVID-19 vaccine status. The third section was the IPAQ questionnaire, and the fourth section was the PSQI questionnaire. These two questionnaires were chosen because they have been shown to have a high reliability and good validity, and are time efficient [8,37,38,39]. The body mass index (BMI) was calculated, based on self-reported data, by dividing the weight (kg) by the height (in m^2^). Participants were then classified into four categories based on their BMI: (i) underweight (<18.5 kg/m^2^), (ii) healthy weight (18.5–24.9 kg/m^2^), (iii) overweight (25.0–29.9 kg/m^2^), and (iv) obese (≥30.0 kg/m^2^) [40].

The IPAQ-SF includes questions regarding the duration spent on vigorous and moderate PA, walking, and sitting over the past seven days. The IPAQ-SF questionnaire has been validated in the adult population of different countries [41,42]. It presents acceptable validity (r = 0.30) and test-retest reliability (r = 0.80) [41]. The total sum score is expressed in metabolic equivalent of task (MET) minutes per day or week. The following values were used for analyzing the IPAQ-SF data: walking = 3.3 METs, moderate PA = 4.0 METs, and vigorous PA = 8.0 METs. Based on the IPAQ responses, participants were classified into three categories (i) a high PA level, if they were engaged in vigorous activity for 3 or more days for at least 1500 MET min per week, or performed 7 days of a combination of walking, moderate intensity, or vigorous intensity activities, achieving 3000 MET min-week; (ii) a moderate PA level, if they met any of the following criteria, performed three or more days of vigorous activity for at least 20 min, performed five or more days of moderate activity/walking for at least 30 min, or performed five or more days of any combination of activities, achieving a minimum of 600 MET min per week; (iii) a low PA level if they did not meet any of the criteria of either the high or moderate levels of PA [12].

The PSQI was used to assess the overall sleep quality over the preceding month. It consists of 18 items, divided into seven sleep-related variables: [1] sleep quality; [2] sleep latency; [3] sleep duration; [4] sleep efficiency; [5] sleep disturbance; [6] medication use; and [7] daytime dysfunction. Every item is rated on a 4-point Likert scale, in terms of frequency or severity. The sum of the component scores yields a global PSQI score ranging from 0 to 21, with higher scores indicating greater sleep disturbance. The PSQI has a strong test–retest reliability (r = 0.87), and good internal consistency (r = 0.80) [38,43]. Participants were classified, based on their PSQI responses, into two categories: (i) poor sleep quality if they had a global score of 5 or less, and (ii) good sleep quality if they had a global score of more than 5 [12].

### 2.3. Statistical Analysis

Statistical analyses were performed using the IBM SPSS software, version 28 (IBM Corp., Armonk, NY, USA). Frequencies with proportions were reported, to describe the characteristics of participants. A chi-squared test (*X*^2^) was used to determine the relationship between the participants’ characteristics, PA levels, and sleep quality. For the primary outcome, the IPAQ and PSQI scales were sorted into two categories, based on their cut-off scores. Variables that were significantly associated with the IPAQ and PSQI in bivariate analyses were included in the multivariate binary logistic regression model. Statistical significance was set at *p* ≤ 0.05.

## 3. Results

A total of 638 adults of both sexes, aged between 18 and 55 years, participated in the study. There were no duplicate (Google form) survey responses. Of the 638 participants in the study, 491 (77%) were women. The mean age (±SD) of all participants was 27.5 (±10.5) years. Table 1 shows the characteristics of the study participants, in frequencies and percentages. Table 2 presents the bivariate analysis for factors associated with physical activity, revealing that sex and nationality were significantly associated with physical activity, *p* ≤ 0.05. Table 3 shows that men are more likely than women to engage in physical activity, odds ratio (OR) 1.66, 95% confidence interval (CI) 1.13–224, *p* = 0.010. Furthermore, non-Arab participants were 1.49 times more active than GCC participants (95% CI 1.02–2.18, *p* = 0.037). The bivariate analysis in Table 4 revealed that age, sex, nationality, and occupation were significantly associated with sleep quality, *p* ≤ 0.05. Table 5 demonstrates that participants over the age of 40 were 1.70, 95% CI 1.01–2.89, *p* = 0.048, more likely to have a good sleep quality, compared to participants under the age of 40. Men showed a better sleep quality, compared to women (OR 1.10, 95% CI 0.69–1.70, *p* = 0.725). Furthermore, non-Arab individuals had a better sleep quality, compared to participants from the GCC (OR 1.95, 95% CI 1.27–2.97, *p* = 0.002). In addition, employed participants showed a better sleep quality than unemployed participants (OR 2.76, 95% CI 1.32–5.76, *p* = 0.007).

**Figure 1:**
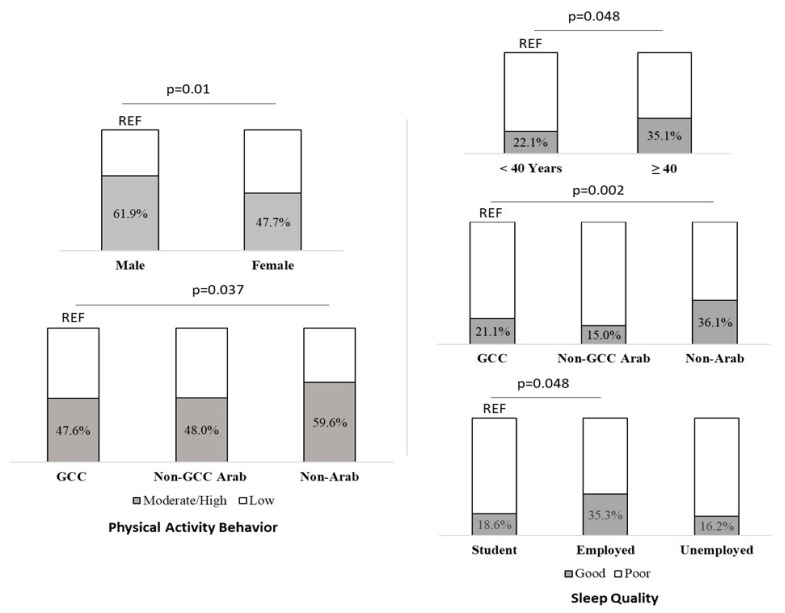
Recapitulative figure of the main moderating variables of physical activity behaviors and sleep quality.

Figure 1 summarizes the key moderating factors of physical activity behaviors and sleep quality. In comparison to 47.7% of women, 61.9% of men were more likely to engage in moderate/high physical activity, *p* = 0.002 (Figure 1). 59.6% of non-Arab participants were more likely to engage in moderate/high physical activity, compared to 47.6% of participants from the GCC, and 48% of non-GCC Arab participants, *p* = 0.029 (Figure 1).

## 4. Discussion

This study investigated the association of sociodemographic factors with PA and sleep quality among adults during the COVID-19 pandemic, by collecting subjective PA and sleep-quality data. The present findings demonstrate that, during the COVID-19 pandemic, there was an association between sociodemographic factors, PA levels, and sleep quality. The findings revealed that PA levels were associated with sex and nationality (GCC, non-GCC Arab, non-Arab). Men engaged in PA more than women, and non-Arab participants were more active, compared to GCC and non-GCC Arab participants. Our findings certainly agree with the compendium on physical inactivity prevalence among 38 Muslim countries [31]. Moreover, a survey of 612 Italians also revealed that women were less active than men (57% vs. 43%) during the COVID-19 lockdown [11]. Almost half of our participants (49.1%) demonstrated a low PA during the COVID-19 pandemic, similar to in a study conducted among university students in the UAE prior to the pandemic [8].

These results are in accordance with findings from a previous cross-sectional study that was conducted in Brazil, to investigate the association between sociodemographic factors and PA and sedentary behaviors in adults with chronic diseases aged 18 years or above, during the COVID-19 pandemic [3]. Indeed, the findings of this study indicated that men were more likely to meet the minimum recommended levels of PA, compared to female participants. A previous study reported a higher rate of PA among men [44]. Men have been reported to be engaged in more PA compared to women during the pandemic, which could be attributed to certain facilitators and barriers to PA [9,45]. Regardless of the pandemic, women have been reported to be less physically active than men in Muslim countries [31]. Possible reasons for a low PA engagement among women in the UAE include cultural or societal barriers, the harsh weather, with high temperatures affecting outdoor PA, restrictive traditional clothing, the increased availability of housemaids, the use of labor-saving devices for household chores, inactive occupations, sedentary leisure time, etc. [32,33]. The study by Da silva et al. (2020) reported that older adults (60 years or older) and those with multimorbidity had a higher level of sedentary behavior (sitting 4 h or more per day), compared to younger (18 to 59 years) participants, but they did not study the association between sociodemographic factors and sleep quality [3].

Our study also showed that non-Arab participants were more active than Arab participants during the COVID-19 pandemic. In general, regardless of the pandemic, it has been reported that Arab people are nearly twice as likely to be physically less active as non-Arab people, based on a compendium on physical inactivity prevalence among 38 Muslim countries [31]. Arab individuals have reported family responsibility and cultural restrictions as reasons for low engagement in PA [46,47]. Donnelly et al. (2018) reported additional barriers to their engagement in PA, such as a lack of time and motivation, pain, and cultural norms [46]. Among the participants included in the study, 108 out of 159 were Arab and had recovered from COVID-19. Whether their COVID-19 status might have affected their PA levels following recovery requires further investigation [48].

Most participants (75.5%) demonstrated a poor sleep quality in our study. Our study also showed that sleep quality was associated with age, sex, nationality, and occupation. The present results showed that participants aged 40 years and above had a better sleep quality than participants younger than 40 years. Men had a better sleep quality than women, and non-Arab participants had a better sleep quality, compared to participants from the GCC, and non-GCC Arab participants.

It has been previously shown that sleep quality generally decreases across a lifespan, with the most significant impact observed on sleep efficiency [24]. Younger adults tend to be more likely than older adults to experience a pattern of sleep problems characterized by poor sleep quality and longer sleep latency, whereas older adults are more prone to inefficient sleeping, marked by extended periods spent in bed without actually being asleep [24]. Additionally, the probability of being a ‘good’ sleeper, unaffected by any adverse sleep symptoms, decreases considerably after the age of 50 [24]. As for the underlying explicative mechanism, the reduced sleep duration and quality with advanced age have been associated with cognitive impairment, as well as with alterations in the brain physiology and structural connectivity [49,50]. Particularly, the white matter microstructure has been shown to underpin the effects of sleep quality [51], as evidenced by the significant positive associations between sleep quality and white matter integrity [52,53]. However, another study did not find evidence for associations between self-reported sleep and neural health, as evidenced by a mostly stable relationship across a lifespan, and the absence of any strong relation (after controlling for age) between sleep quality and neural health (i.e., fractional anisotropy) above and beyond old age [24]. Sleep quality may also be altered with age, due to changes in the mental health conditions, as well as the use of sleep medication [54]. Taken together, age-related changes in sleep patterns appear to be complex and multifaceted, and warrant further experimental neurophysiological research.

In a study conducted during COVID-19 in Turkey, among university students aged (18–30 years), male students reported higher PA levels and a better sleep quality, compared to female students. However, this study only investigated the effect of COVID-19 confinement on PA and sleep quality, with no focus on their association with the demographic characteristics of the students [55]. Another study on university students (aged from 19 to 27 years) in Saudi Arabia found that male students were more physically active than female students, based on self-reported IPAQ scores. However, the study showed no significant differences in sleep quality (PSQI scores) between male and female students [56]. Likewise, Romdhani et al. (2021) found higher PSQI scores in female athletes, which indicates a poor sleep quality [57].

A higher prevalence of depression, vulnerability to stress, and emotional exhaustion in women, and sex-based differences in the biology of sleep, could also be some of the reasons for the differences in sleep quality between men and women [9,34,35]. Moreover, employed participants reported better sleep quality, in comparison with unemployed participants and students. This is supported by a study by Hyun et al. (2021), wherein young adults demonstrated a poor sleep quality, due to disruptions to work and school, such as remote work or job loss [58].

The PA and sleep-quality patterns of most participants in our study were relatively low during the COVID-19 pandemic, which could possibly be due to travel restrictions and home confinement, due to the COVID-19 lockdown. Similarly, a significant reduction in PA levels, and an increase in sleeping time were noticed in a study by Sanudo et al. (2020) [59]. The poor PA could be attributed to the following factors: the closure of gyms, a drastic change in everyday schedules and habits, and the movement restrictions imposed during the lockdown period [12,14,60]. The poor sleep patterns could occur due to increased anxiety and worried pre-sleep thoughts because of the pandemic, the negative effects of the infection, and financial or job security concerns [12,14,19].

One in three participants demonstrated lower PA levels during the COVID-19 lockdown in the UAE [61]. Among UAE residents, Cheikh et al. (2020) reported that 38.5% participants reported not engaging in PA, 28.1% had a poor sleep quality, and 60.8% had sleep disturbances, during the pandemic [62]. The negative effects of low PA levels and poor sleep quality, in addition to increased risk factors for chronic diseases, and lower immunity, might have resulted in more severe symptoms and worse health outcomes against infection during the COVID-19 pandemic [62]. There seems to be a strong association between PA levels and sleep quality, and better mental health scores, which warrants further investigation in Arab populations.

The main strength of this study is the relatively large sample size recruited, which was considered a very good sample size at over 500, based on Rahi et al. (2019) [63]. Based on the final sample size of our study of 638 participants, the proportion (prevalence) of our study group for good sleep quality of 24.5% compared to the population proportion (prevalence) of 50% (used to calculate the sample size initially) with a probability of type I error of 5%, the post-hoc power is 100%. Our study addressed the association of multiple sociodemographic and anthropometric variables with PA and sleep quality in the UAE.

There were no objective measures used along with the self-reported questionnaires in our study, although the IPAQ has been validated among different age groups in many countries [64]. Moreover, the PSQI doesn’t assess nap duration; therefore, the sleep quality scores of an individual may be affected, given that daily naps are well-known for their valuable effect on health [65]. Additionally, the use of these self-reported measures may give rise to potential biases, because of participants’ social desirability, recall period, or selective recall (of only certain experiences) [66]. Lastly, as pre-pandemic PA and sleep quality data were not collected, we were unable to determine whether participants had similar PA levels and sleep quality before and during the COVID-19 pandemic.

## 5. Conclusions

Two or more sociodemographic factors, such as age, sex, nationality, and occupation have been found to be associated with self-reported PA levels and/or sleep quality in individuals with and without COVID-19 during the COVID-19 pandemic. Male and non-Arab participants seem to be the less affected populations, in terms of both PA and sleep quality, compared to female and Arab participants, during the COVID-19 pandemic. Therefore, strategies to promote PA and enhance sleep quality in women and in Arab populations are further warranted.

## Figures and Tables

**Table 1:** Characteristics of the study participants, n = 638.

Variable	n
**Age, years**	
<40	**542**
≥40	114
**Gender**	
Female	**491**
Male	147
**Nationality**	
GCC	**370**
Non-Arab	166
Non-GCC Arab	100
**BMI**	
Underweight	50
Normal	**317**
Overweight	172
Obese	97
**Occupation**	
Student	**338**
Employed	232
Unemployed	68
**Number of people per room**	
1–2	76
3–5	236
>5	**326**
**Number of people per room**	
1–2	**543**
≥3	95
**History of smoking**	
Yes	68
No	**570**
**COVID-19 status**	
Recovered	149
Not infected	**256**
**Physical activity category**	
Low	313
Moderate/high	**325**
**Sleep quality**	
Poor	**482**
Good	156

Proportions in bold are the highest; GCC, Gulf Cooperation Council; BMI, body mass index.

**Table 2:** Bivariate analysis between physical activity and participants’ characteristics, using chi-squared tests.

Variable	Physical Activity, n (%)		*p* Value
	Low	Moderate/High	
**Age, years**			
<40	253 (48.3)	271 (51.7)	0.400
≥40	60 (51.6)	54 (47.4)	
**Sex**			
Female	257 (52.3)	234 (47.7)	**0.002**
Male	56 (38.1)	91 (61.9)	
**Nationality**			
GCC	194 (52.4)	176 (47.6)	
Non-GCC Arab	52 (52)	48 (48)	**0.029**
Non-Arab	67 (40.4)	99 (59.6)	
**BMI**			
Underweight	28 (56)	22 (44)	
Normal	152 (47.9)	165 (52.1)	0.391
Overweight	79 (45.9)	93 (54.1)	
Obese	53 (54.6)	44 (45.4)	
**Occupation**			
Student	176 (52.1)	162 (47.9)	
Employed	100 (43.1)	132 (56.9)	0.071
Unemployed	37 (54.4)	31 (45.6)	
**History of smoking**			
Yes	32 (47.1)	36 (52.9)	0.727
No	281 (49.3)	289 (50.7)	
**COVID-19 status**			
Yes	74 (49.7)	75 (50.3)	0.866
No	239 (48.9)	250 (51.1)	
**Sleep quality**			
Poor	245 (50.8)	237 (49.2)	0.116
Good	68 (43.6)	88 (56.4)	

*p* values in bold are statistically significant; GCC, Gulf Cooperation Council; BMI, body mass index.

**Table 3:** Binary logistic analysis of the predictors of physical activity.

Variable	uaOR (95% CI)	aOR (95% CI)	*p* Value
**Sex**			
Female (Reference, 1)			
Male	1.79 (1.22, 2.60)	1.66 (1.13–2.44)	**0.010**
**Nationality**			
GCC (Reference, 1)			
Non-Arab	1.63 (1.12, 2.36)	1.49 (1.02–2.18)	**0.037**
Non-GCC Arab	1.02 (0.65, 1.58)	0.97 (0.62–1.51)	0.896

*p* values in bold are statistically significant; GCC, Gulf Cooperation Council; uaOR, unadjusted odds ratio; aOR, adjusted odds ratio; CI, confidence interval.

**Table 4:** Bivariate analysis between sleep quality and participants’ characteristics, using chi-squared tests.

Variable	Sleep Quality, n (%)		*p* Value
	Good	Poor	
**Age, years**			
<40	116 (22.1)	408 (77.9)	**0.004**
≥40	40 (35.1)	74 (64.9)	
**Sex**			
Female	109 (22.2)	382 (77.8)	**0.016**
Male	47 (32)	100 (68)	
**Nationality**			
GCC	81 (21.1)	289 (78.9)	
Non-GCC Arab	15 (15)	85 (85)	**<0.001**
Non-Arab	60 (36.1)	106 (63.9)	
**BMI**			
Underweight	12 (24)	38 (76)	
Normal	84 (26.5)	233 (73.5)	0.705
Overweight	38 (22.1)	134 (77.9)	
Obese	22 (22.7)	75 (77.3)	
**Occupation**			
Student	63 (18.6)	275 (81.4)	
Employed	82 (35.3)	150 (64.7)	**<0.001**
Unemployed	11 (16.2)	57 (83.8)	
**Number of people per room**			
1–2	133 (24.5)	410 (75.5)	0.953
≥3	23 (24.2)	72 (75.8)	
**History of smoking**			
Yes	12 (17.6)	86 (82.4)	0.167
No	144 (25.3)	426 (74.7)	
**COVID-19 status**			
Yes	124 (25.4)	365 (74.6)	0.335
No	32 (21.5)	117 (78.5)	
**Physical activity**			
Low	68 (21.7)	245 (78.3)	0.116
Moderate/High	88 (27.1)	237 (72.9)	

*p* values in bold are statistically significant; GCC, Gulf Cooperation Council.

**Table 5:** Binary logistic analysis of the predictors of a good sleep quality.

Variable	uaOR (95% CI)	aOR (95% CI)	*p* Value
**Age, years**			
<40 (Reference, 1)			
≥40	1.90 (1.23, 2.94)	1.70 (1.01–2.89)	**0.048**
**Gender**			
Female (Reference, 1)			
Male	1.65 (1.10, 2.47)	1.10 (0.69–1.70)	0.725
**Nationality**			
GCC (Reference, 1)			
Non-Arab	2.02 (1.35, 3.02)	1.95 (1.27–2.97)	**0.002**
Non-GCC Arab	0.63 (0.35, 1.15)	0.60 (0.32–1.11)	0.101
**Occupation**			
Unemployed (Reference, 1)			
Employed	2.83 (1.41, 5.70)	2.76 (1.32–5.76)	**0.007**
Student	1.19 (0.59, 2.39)	1.47 (0.68–3.18)	0.329

*p* values in bold are statistically significant; GCC, Gulf Cooperation Council; uaOR, unadjusted odds ratio; aOR, adjusted odds ratio; CI, confidence interval.

## Data Availability

The data presented in this study are available on request from the corresponding author.
